# Correlation of gastrointestinal perforation location and amount of free air and ascites on CT imaging

**DOI:** 10.1007/s00261-021-03128-2

**Published:** 2021-06-10

**Authors:** Dionysios Drakopoulos, Jacqueline Arcon, Peter Freitag, Mostafa El-Ashmawy, Steven Lourens, Guido Beldi, Verena Carola Obmann, Lukas Ebner, Adrian Thomas Huber, Andreas Christe

**Affiliations:** 1 Department of Diagnostic, Interventional and Pediatric Radiology, INSELGROUP, Radiology Division SLS, University of Bern, Tiefenaustrasse 112, 3004 Bern, Switzerland; 2 Department of Visceral and Transplantation Surgery, Inselspital, Bern University Hospital, University of Bern, Freiburgstrasse 10, 3010 Bern, Switzerland; 3 Department of Diagnostic, Interventional and Pediatric Radiology, Inselspital, Bern University Hospital, University of Bern, Freiburgstrasse 10, 3010 Bern, Switzerland

**Keywords:** Gastrointestinal perforation, Abdominal CT imaging, CT volumetry, Volumetry of ascites, Volumetry of air

## Abstract

**Purpose:**

To analyze the amount of free abdominal gas and ascites on computed tomography (CT) images relative to the location of a perforation.

**Methods:**

We retrospectively included 172 consecutive patients (93:79 = m:f) with GIT perforation, who underwent abdominal surgery (ground truth for perforation location). The volume of free air and ascites were quantified on CT images by 4 radiologists and a semiautomated software. The relation of the perforation location (upper/lower GIT) and amount of free air and ascites was analyzed by the Mann–Whitney test. Furthermore, best volume cutoff for upper and lower GIT perforation, areas under the curve (AUC), and interreader volume agreement were assessed.

**Results:**

There was significantly more abdominal ascites with upper GIT perforation (333 ml, range 5 to 2000 ml) than with lower GIT perforation (100 ml, range 5 to 2000 ml, *p* = 0.022). The highest volume of free air was found with perforations of the stomach, descending colon and sigmoid colon. Significantly less free air was found with perforations of the small bowel and ascending colon compared to the aforementioned. An ascites volume > 333 ml was associated with an upper GIT perforation demonstrating an AUC of 0.63 ± 0.04.

**Conclusion:**

Using a two-step process based on the volumes of free air and free fluid can help localizing the site of perforation to the upper, middle or lower GI tract.

**Graphic abstract:**

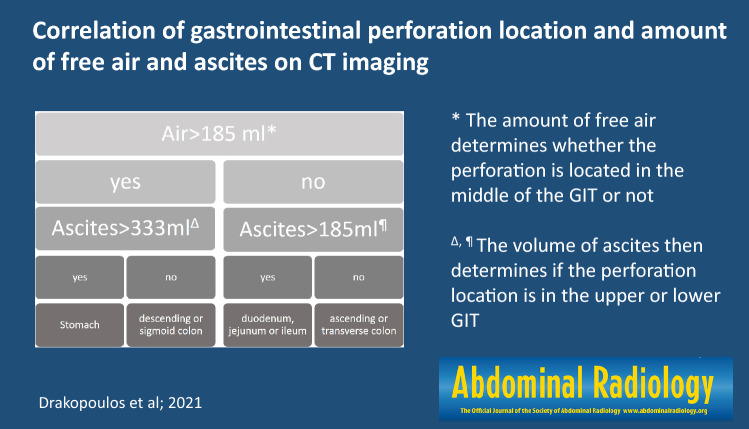

## Introduction

Breaching of the gastrointestinal (GI) tract wall can be due to ulcer disease, inflammatory disease, blunt or penetrating trauma, iatrogenic factors, a foreign body or a neoplasm [1–6]. The most important questions to be answered regard the identification of the presence, location, and cause of the perforation in order to perform the appropriate therapeutic procedure because gastrointestinal tract (GIT) perforation is a major life-threatening condition with high morbidity and mortality that requires emergency surgery; despite improvements in surgical and medical treatments, the overall mortality rate is 30%, and the mortality rate of cases that also involve diffuse peritonitis is up to 70% [7–10]. Clinical diagnosis of the site of GIT perforation is difficult, as the symptoms may be nonspecific. The clinical presentation varies; esophageal perforations can present with acute chest pain, odynophagia and vomiting, gastroduodenal perforations cause acute, severe abdominal pain, while colonic perforations tend to follow a slower course of progression with secondary bacterial peritonitis or localized abscesses.

The presence of free intraperitoneal gas on a routine radiograph usually indicates bowel perforation. Some studies have shown that as little as 1 ml of gas can be detected below the right hemidiaphragm on properly exposed erect chest radiographs [11]. Plain film radiography (erect chest and abdominal radiographs) is sensitive in only 50–70% of cases, and the site of perforation is almost never elucidated [12, 13]. A left lateral decubitus film can also be used for the detection of small amounts of free air that may be interposed between the free edge of the liver and the lateral wall of the peritoneal cavity. When interpreting a right lateral decubitus image, gas within the stomach or colon may obscure small amounts of free air. Other modalities include ultrasound, which may be particularly useful in patient groups where the radiation burden should be limited, notably children and pregnant women. However, ultrasound should not be considered a first choice in excluding pneumoperitoneum [14]. Computed tomography (CT) is useful in detecting minute amounts of extraluminal gas [15, 16], the sensitivity of CT for free gas lies between 92 and 100% [17–20] A study of multidetector CT showed 86% accuracy in identifying the site of perforation [21].

Time is of the essence in these patients. Knowing the exact location of GIT perforation is crucial for surgeons, since the operation time, as well as morbidity and mortality, can thereby be decreased.

In this study, we aimed to predict the location of perforations by analyzing the amount of free abdominal gas and ascites on CT images. Our hypothesis was that more free gas than ascites in the abdomen indicates an upper GIT perforation; and more ascites than gas indicates a lower GIT perforation due to peritonitis.

## Materials and methods

The Cantonal Ethics Committee approved this retrospective study (Ethics Approval Nr. 2020-01279).

### Recruitment

A full-text search for “perforation” in the radiological information system (RIS, GE Healthcare, Chicago, Illinois, USA) was performed between 01.01.2003 and 01.01.2020 by a PhD student. Patients who had been examined by abdominal CT in our emergency room with or without the use of contrast media and whose records included the word “perforation” in the radiological report were included.

Exclusion criteria:

1. Patients without available operation report were excluded (not operated on, not operated in our hospital).
2. Patients with no perforation in the radiological report were excluded (“suspicion of perforation” or “no signs of perforations”, both showed in the radiological full-text search).
3. Perforation with obvious perforation locations like covert perforations or extraperitoneal perforations were excluded.
4. Patients with other reasons for free air than GIT perforation were excluded like postinterventional (drainage) or postsurgical free gas; but also posttraumatic patients with perforating injuries (abdominal wall laceration/defect).
5. Patients with other reasons for ascites than GIT perforation were excluded, like liver cirrhosis, peritoneal carcinomatosis, pancreatitis and trauma with hemorrhagic ascites (trauma patients with hyperdense ascites were excluded, nontrauma patients with hyperdense ascites (blood or contrast) were included).

**Fig. 1:**
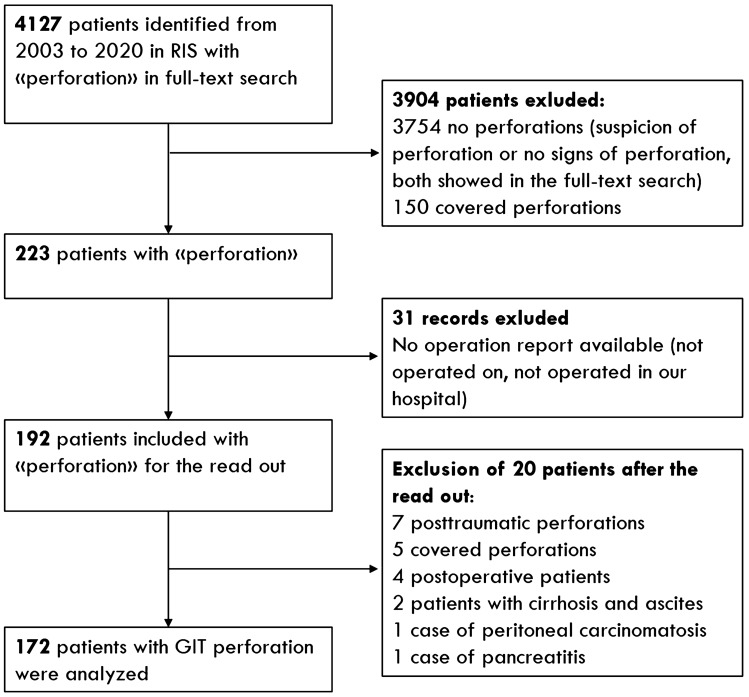
Flowchart for inclusion and exclusion of patients, preferred reporting items for systematic analyses (PRISMA). Covered perforations were excluded: the perforation is sealed off by adjacent organs, but the perforation location is obviously revealed by small air bubbles in close neighborhood of the perforation

A total of 223 patients were found and matched to the surgical operation report from the CGM CLINICAL clinical information system (CompuGroup Medical Schweiz AG, Bern, Switzerland, Version: 7.16.1-5). The location of the perforation was extracted from the operation reports and represented the ground truth. After applying the exclusion criteria 172 patients remained for our study (Flowchart Fig. 1). The matching CT images were selected and anonymized by the PhD student and transferred to a read-out folder in our picture archiving and communication system (PACS, IDS7, Sectra, Linköping, Sweden). Consecutive case numbering was assigned to each patient.

### Image acquisition

Two different CT scanners (Siemens SOMATOM Sensation 16 and SOMATOM Definition Edge, both from Siemens Healthcare, Erlangen, Germany) were used. Before 2012, the older model SOMATOM sensation 16 applied 120-140 kVp, 160 reference mAs tube current, 16 × 0.75-mm collimation, 1.15 pitch and standard filtered back projection with a slice thickness of 5 and 2 mm. A total of 120 ml of standard iodinated contrast medium (CM) with 300 mg/ml iodine (iobitridol, Xenetix 300; Guerbet, Aulnay-sous-Bois, France) was administered intravenously (i.v.) with an image acquisition delay of 60 s and a flow rate of 3 ml/s. Telebrix gastro with an iodine concentration of 300 mg/ml (Megluminioxitalamat, Guerbet, Aulnay-sous-Bois, France) was used as oral and rectal CM: 24 ml CM was dissolved with 800 ml tap water. This CM was orally administered 1 h prior to the CT exam and instilled rectally directly before the scan.

Since 2012, the new SOMATOM Definition Edge has used 100–140 care kVp, 120–160 reference mAs as the tube current, 128 mm × 0.6 mm collimation, 0.6 pitch and iterative reconstruction (SAFIRE, level 3). Transverse images were reconstructed at intervals of 5 and 1 mm. One hundred milliliters of Iomeron 400 mg/ml was injected intravenously with a flow rate of 3 mL/s. Data acquisition was started after 70 s. No oral or rectal CM was used anymore. Before 2012 the gold standard in emergency abdominal CT imaging was IV, oral and rectal CM application [22, 23]. To save time for the critically ill patients our department changed the emergency CT protocols in 2012 to IV CM without oral or rectal CM application [24–26].

### Image analysis

**Fig. 2:**
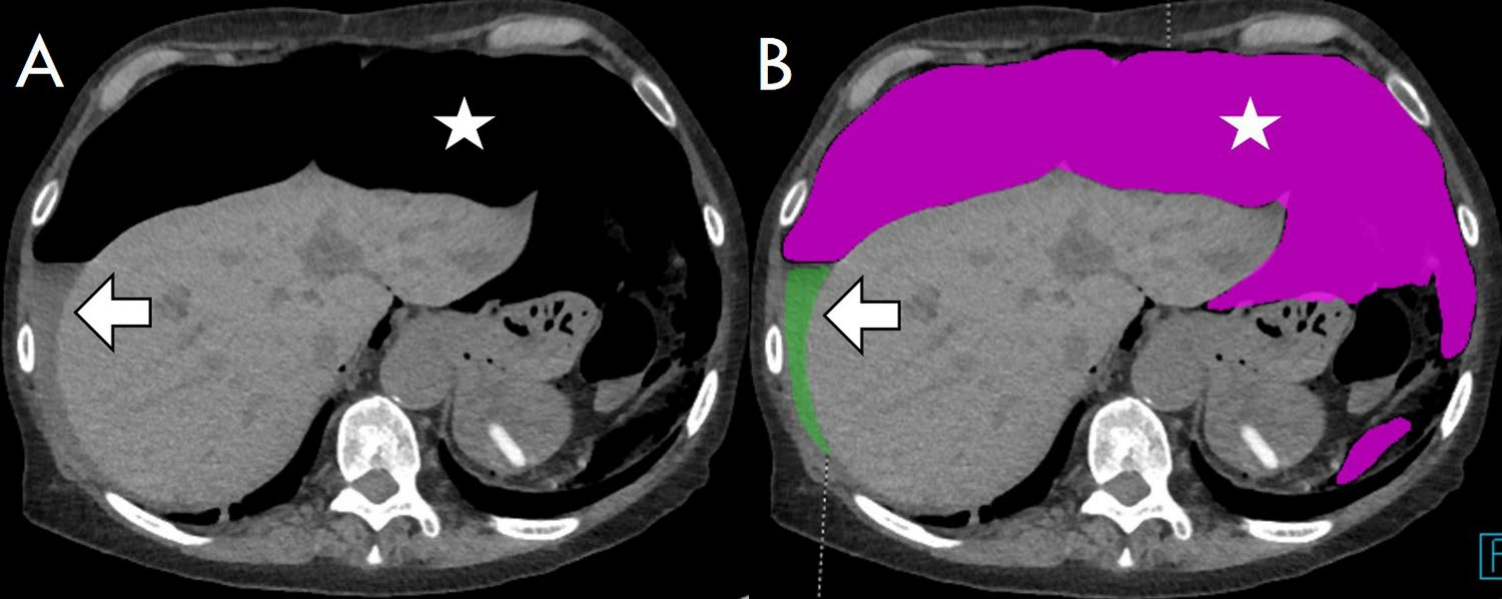
Semiautomated volumetry on axial slices of a perforation in the descending colon. **A** Preannotation and **B** postannotation of free air (asterisk, pink) with a total volume of 1972 ml and 93 ml of ascites (arrow, green)

Images were reviewed by 4 board-certified radiologists (designated as 1, 2, 3, and 4) with 26, 33, 9, and 22 respective years of experience in abdominal imaging. Radiologists 1 and 2 analyzed 100 cases separately and radiologists 3 and 4 read 92 cases separately (different from the first 100 cases). All four readers were blinded from each other and to the perforation location. During the read out, a total of 20 patients had to be excluded (e.g., posttraumatic, postoperative and covert perforations, details in Flowchart of Fig. 1) leading to the remaining 172 study patients. The average volume of the paired readers was calculated for free air and ascites:

The amount of free air and ascites was rated by visual comparison to the volume of cooking or drinking units: teaspoon (5 ml), tablespoon (15 ml), shot glass (40 ml), small drinking glass (1 dl = 100 ml), soda can (333 ml), and PET bottle (500 ml/1000 ml/1500 ml/2000 ml; PET: PolyEthylene Terephthalate plastic bottle). Two additional medical technicians from our imaging laboratory performed a semiautomated computer-aided volume measurement of free air and ascites from 35 of the first 100 cases and 30 of the following 92 patients using syngo.via (Siemens Healthcare GmbH, Erlangen, Germany). Semiautomated means that the software uses a region-growing process that suggests the area of free air/ascites on the CT scan. The 3D annotation requires a time-consuming manual adjustment by the medical technician to differentiate air from feces and fat (or ascites from soft-tissue structures) (Fig. 2). On average the 3D analysis of one patient took 30 min. Because of that time constraint we planned the 3D analysis only in one third of the patients, randomly. The semiautomated measurement was used for detecting accuracy and agreement of radiologists in estimating the volume of ascites and free air.

The human raters determined if there was more gas than ascites to test our hypothesis for an easy applicable tool without performing measurements by the radiologists. Furthermore, they estimated the most likely location of the perforation by using radiological criteria (most extensive free gas or air bubble accumulation, bowel wall thickening or discontinuation and most extensive mesenteric fat imbibition). They classified the perforation location as follows: (1) stomach, (2) duodenum, (3) jejunum, (4) ileum, (5) ascending colon, coecum or appendix, (6) transverse colon, (7) descending colon and (8) sigmoid colon or proximal rectum. In addition, the radiologists had to indicate their confidence level in rating the perforation location: 1 = no confidence in localizing the perforation site; 2 = some confidence; 3 = 50%:50% confidence, 4 = reasonably sure, 5 = 100% sure of the location.

For each reader, a master read-out Excel file was compiled and saved daily with a traceable calendar date on a server drive with restricted access for radiologists only. For each reader, a personal encoded read-out Excel file was compiled, containing only the patient code and the read-out variables (volume and location). The Excel sheets were stored in SharePoint of our hospital domain.

### Statistics

The label “air scenario” was attributed to cases with more free air than ascites, and the “ascites scenario” was attributed to patients with more ascites than free air. Perforation locations in the (1) stomach, (2) duodenum, (3) jejunum, (4) ileum, (5) ascending colon, coecum or appendix, (6) transverse colon, (7) descending colon and (8) sigmoid colon or proximal rectum were pooled into upper GIT (1–4) and lower GIT (5–8) perforations. The prevalence of the perforation location was analyzed per GIT segment (1–8) by chi square testing. The median absolute volumes of air and ascites for perforations of each GIT segment were calculated by using the average volume estimates of both radiologists. Comparisons among the different segments were performed by using the rank sum test (Mann–Whitney independent testing). The volume of free air and ascites alone and the sum, the delta and the ratio of air and ascites were tested to classify the perforation location as upper or lower GIT using the rank sum test (Mann–Whitney independent testing). The “air scenario” and “ascites scenario” were analyzed by chi square testing as a sign of upper or lower GIT perforation. Receiver operating characteristic (ROC) statistics for the best volume cutoff for upper and lower GIT perforation were calculated, delivering individual sensitivities, specificities and areas under the curve (AUCs). Furthermore, interreader agreement was assessed for volume rating among the four radiologists, and the correlation coefficient and limits of agreement between the semiautomated and radiologist measurements were calculated. Due to the volume approach by drinking units used by the radiologists, the number of entries was limited, and the weighted kappa could be calculated. For the volume comparison with the machine (continuous volume spectrum), the correlation coefficient and the Bland–Altman agreement approach were used. For the following kappa classification of interrater agreement, *κ* < 0 = poor agreement, 0.0–0.20 = slight, 0.21–0.40 = fair, 0.41–0.60 = moderate, 0.61–0.80 = substantial, and 0.81–1.00 = (almost) perfect agreement was used. MedCalc (Version 7.6.0.0, Ostend, Belgium) was used for the statistical computation. A significance level of *p* < 0.05 was applied. The radiologists’ correct perforation location was expressed as the sensitivity for segmental classification and for upper/lower GIT classification. Furthermore, the radiologists’ experience was compared for correct perforation location prediction (chi square testing).

## Results

Between 01.01.2003 and 01.01.2020, a total of 172 abdominal cases with GIT perforations were finally analyzed. The median age was 66.1 years (range 1.2 to 94.4 years), and the sex ratio was 93:79 = m:f. In 5% of our entire study population, CT scans were performed without IV contrast media. Thirty percent, 45% and 25% of the study patients received oral, both oral and rectal and no GIT contrast media, respectively.

### Location of the GIT perforation (consecutive study)

**Fig. 3:**
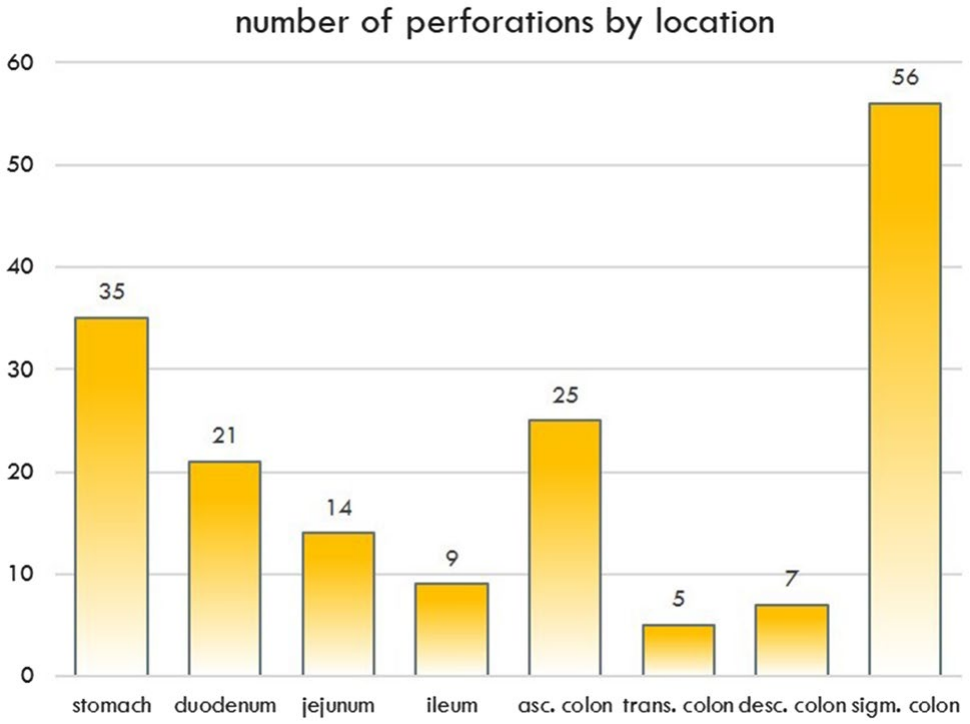
Perforation by location

A total of 54.1% (*n* = 93) of all perforations were found in the upper GIT, and 45.9% (*n* = 79) were found in the lower GIT. The top three locations were the sigmoid colon, stomach and ascending colon, with prevalences of 32.6% (*n* = 56), 20.3% (*n* = 35) and 14.5% (*n* = 25), respectively. The transverse colon demonstrated significantly fewer perforations than the sigmoid colon (2.9%, *p* < 0.0001, entire prevalence statistics shown in Fig. 3 and Table 1).

**Table 1:** Distribution of perforations with air and ascites volume (ml)

PERFORATION			AIR (ml)			ASCITES (ml)		
	*N*	*N*(%)	Median	Percentiles		Median	Percentiles	
				25%	75%		25%	75%
STOMACH	35	20.3	333	119	500	417	100	750
DUODENUM	21	12.2	70	40	225	333	58	750
JEJUNUM	14	8.1	61	23	100	85	23	1500
ILEUM	9	5.2	40	5	93	250	68	625
ASC. COL	25	14.5	40	16	217	70	33	249
TRANS. COL	5	2.9	100	54	750	100	55	1063
DESC. COL	7	4.1	333	21	604	70	57	100
SIGMA/RECT	56	32.6	143	40	500	180	28	333
upper GIT	79	45.9	174	40	417	333	70	750
lower GIT	93	54.1	100	28	500	100	31	333

*N* number of patients

### Dependency of the perforation location and the amount of free air

**Fig. 4:**
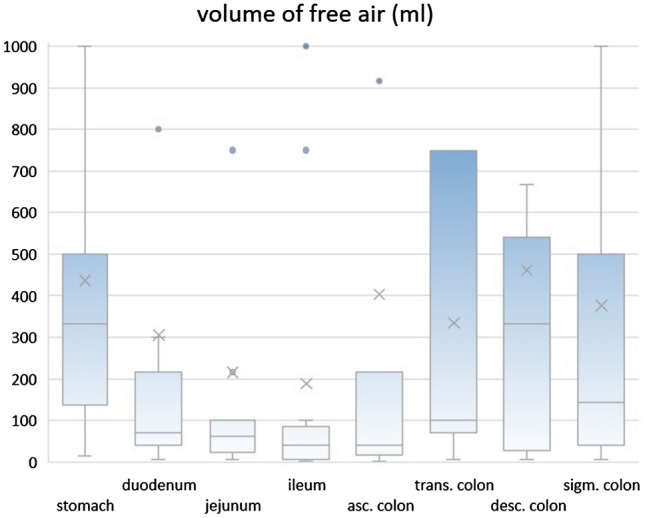
Box-and-Whisker plot of the amount of free air per perforation location

**Fig. 5:**
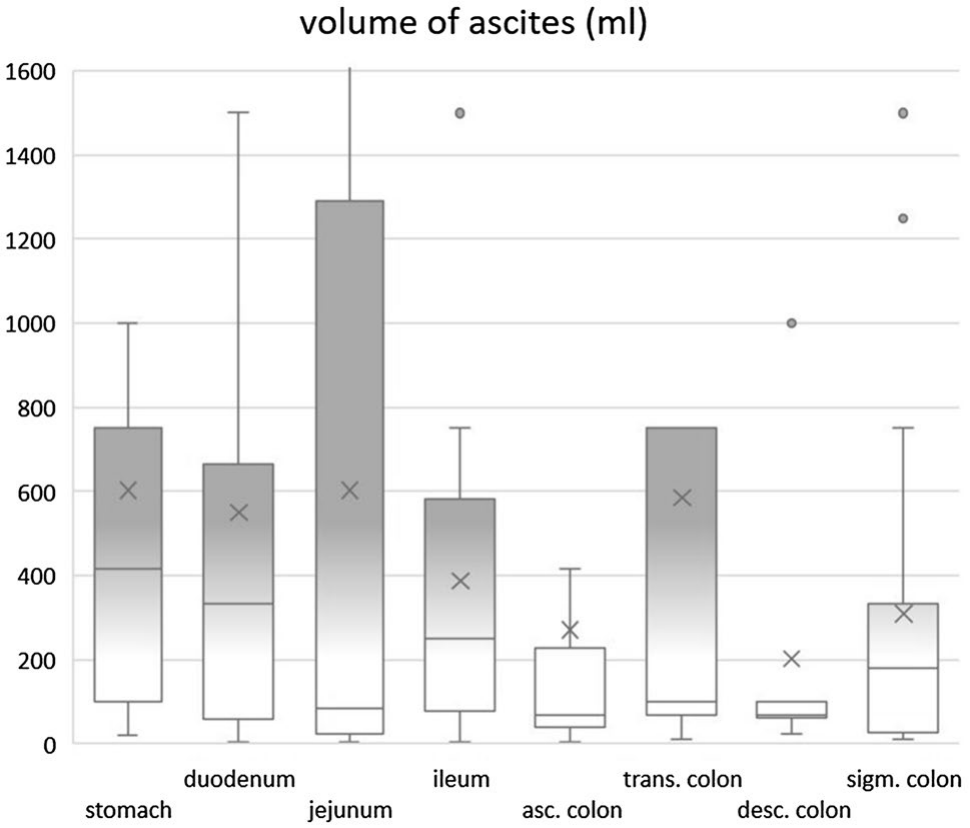
Box-and-Whisker plot of the amount of ascites per perforation location

All of the patients demonstrated free air since this was an inclusion criterion. The median volume of free air was 174 ml (percentiles 25–75% = 40–417 ml) in perforations of the upper GIT, compared to 100 ml (percentiles 25–75% = 28–500 ml) in perforations of the lower GIT (*p* = 0.47). The highest volume of free air was found in perforations of the stomach, descending colon and sigmoid colon (333, 333, 143 ml, entire statistics in Table 1, Fig. 4 and 5). Significantly less free air was present in perforations of the middle GI tract: duodenum, jejunum, ileum ascending and transverse colon leaked only 70, 61, 40, 40 and 100 ml (*p* value: 0.004, 0.0035, 0.001, 0.001 and 0.017, compared to perforations of the stomach).

### Dependency of perforation location and amount of ascites

**Fig. 6:**
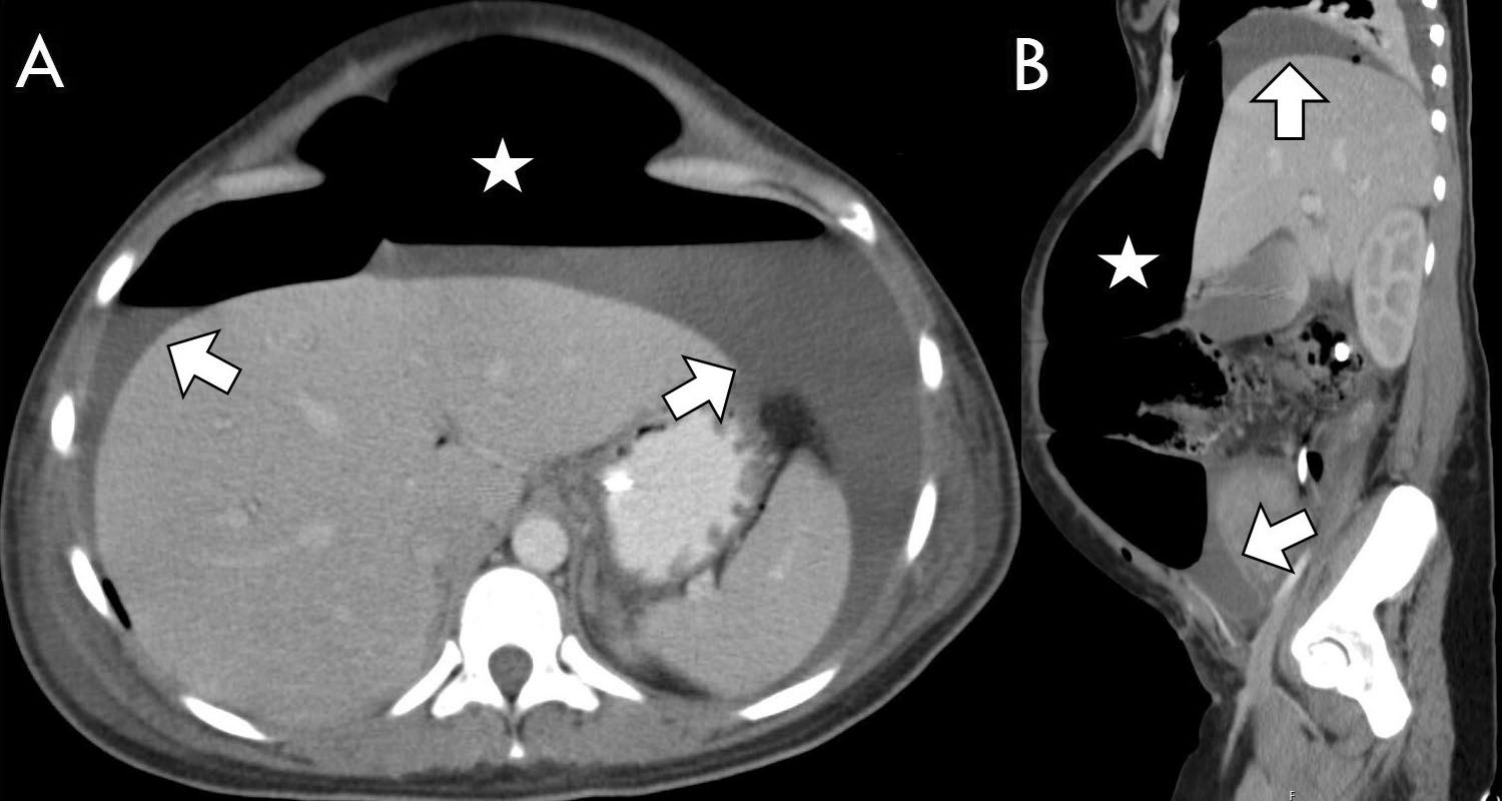
A 32-year-old Woman with a Perforation of the Stomach. Yielding typically large amounts of free air (asterisk) and ascites (arrows). Perforation captured on axial slice (**A**) and on sagittal reformat (**B**)

**Fig. 7:**
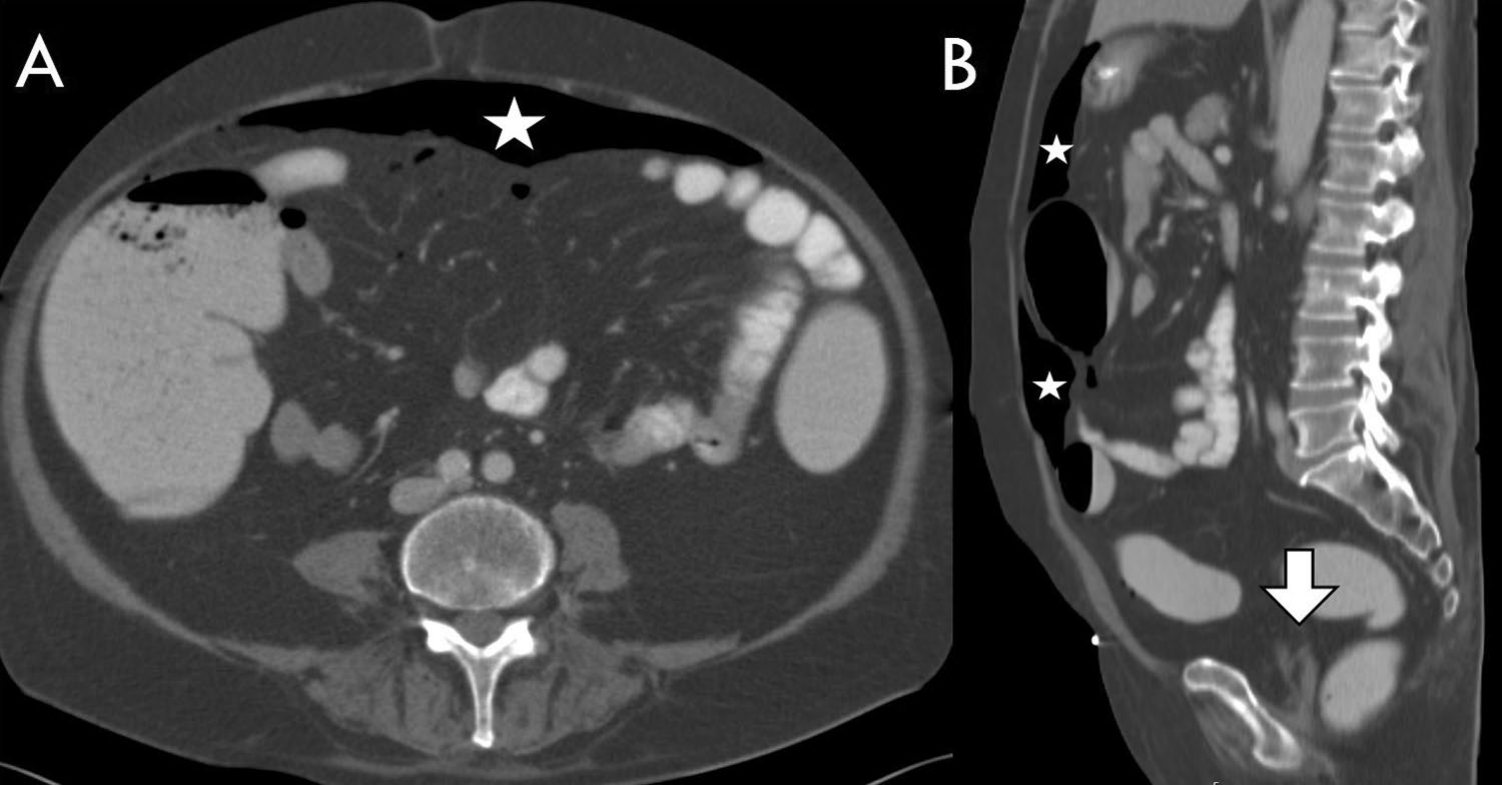
A 58-year-old man with a perforation of the descending colon. Yielding a typically large amount of free air (asterisk) and only a small volume of ascites (arrow). Perforation captured on axial slice (**A**) and on sagittal reformat (**B**)

All of the study patients exhibited a certain amount of ascites (100% prevalence). There was significantly more ascites in the abdomen when the perforation was located in the upper GIT (median: 333 ml, percentiles 25–75% = 70-750 ml) than when it was located in the lower GIT (median 100 ml, percentiles 25–75% = 31-333 ml, *p* = 0.022). Most ascites was found with perforations of the stomach, duodenum and ileum (median: 417, 333, 250 ml, entire statistics in Table 1 and Fig. 5, typical examples in Fig. 6). The presence of significantly less ascites indicated perforations of the large bowel (Fig. 7), e.g., a perforation in the ascending colon yielded an ascites volume of only 70 ml (*p* = 0.004, compared to a perforation of the stomach).

### Relationship of free air and ascites dependent on the perforation location

**Fig. 8:**
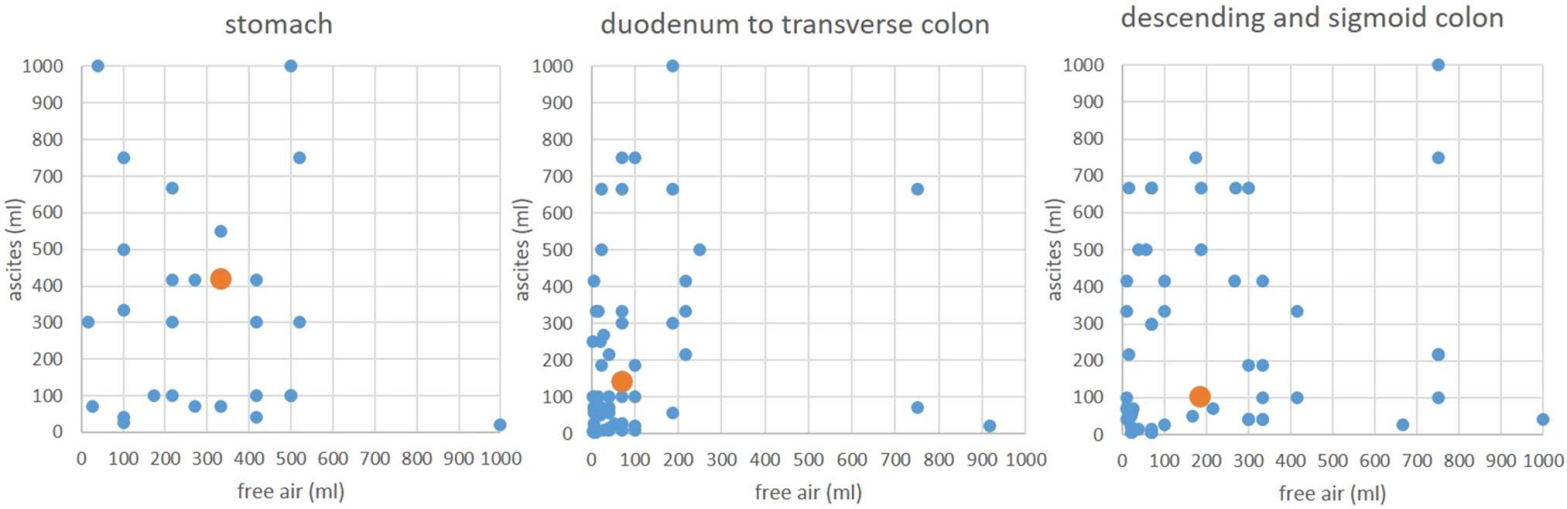
Scatterplot of the volume of free air versus the volume of ascites per location: perforations of the stomach demonstrated the largest volume of free air and ascites (median is shown as orange point), while perforations of the middle GIT lead to smaller volumes (median in orange). Leakage of the descending and sigmoid colon demonstrated more free air than ascites (median in orange). Only volumes smaller than 1000 ml are shown

The delta of air and ascites volumes (*V*_air_ − *V*_asc_) and the sum of both volumes also demonstrated significant differences between the upper and lower GITs (*p* = 0.005 and *p* = 0.037). However, the *p* value of the ascites difference in upper and lower GIT perforations alone was lower (*p* = 0.0023). On the scatterplot diagrams the relation between free air and ascites is shown dependent on the perforation location (Fig. 8). In addition, the volume ratio of air and ascites between the upper and lower GIT perforations or the fact that there was more ascites than air (ascites scenario) did not lead to significant differences (*p* = 0.18, *p* = 0.31).

### ROC analysis of ascites and air

**Fig. 9:**
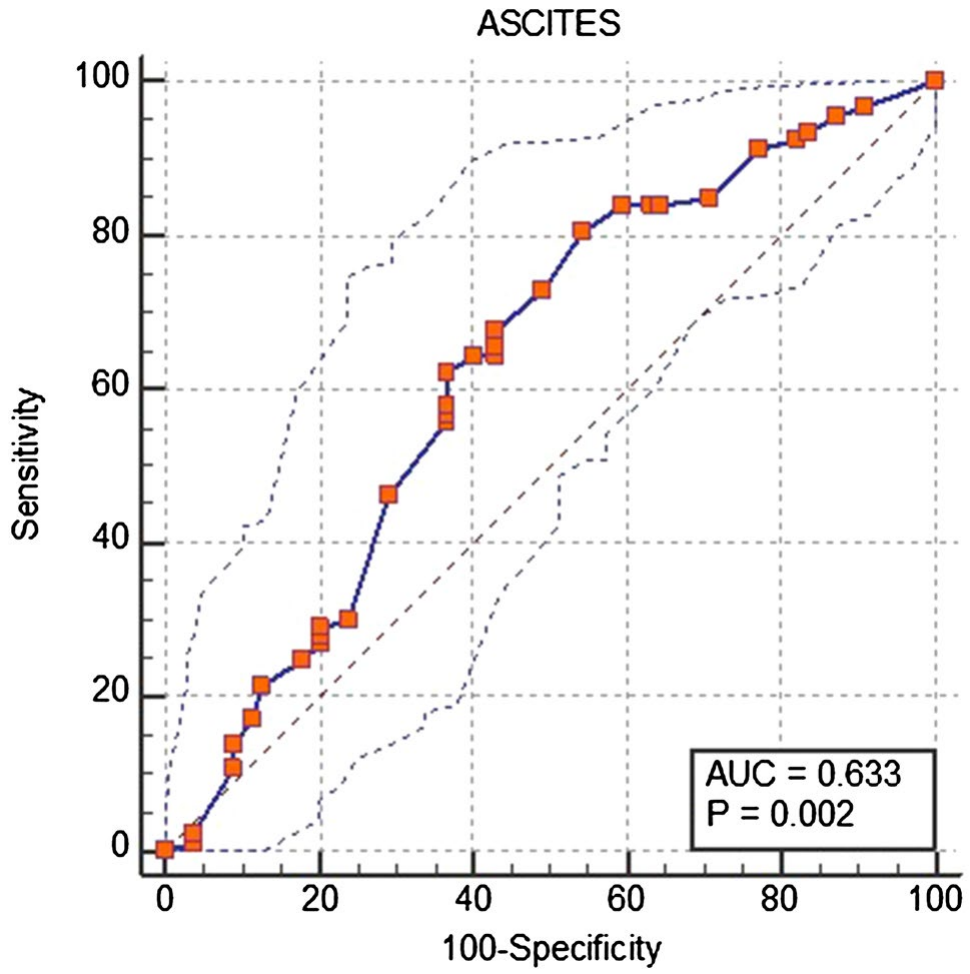
ROC, AUC, sensitivity and specificity of ascites for differentiating upper from lower GIT perforation

The AUC was 0.63 ± 0.04 using the amount of ascites (ml) for differentiating the perforation location (upper vs lower GIT, Fig. 9). A cutoff level of 333 ml ascites was the best criterion for location detection. When a perforation (free air) presented with 333 ml ascites or less, it was more likely a lower GIT perforation (large bowel). The odds ratio for a perforation in the lower GIT when demonstrating less than 333 ml ascites was 3.52 (95% CI 2.7–4.0). This threshold led to a sensitivity and specificity for large bowel perforation of 80.7% and 45.6%, respectively.

Using the volume of free air, a cutoff value of 70 ml or less indicated a lower GIT perforation. However, the odds ratio for a perforation in the lower GIT with less than 70 ml free air was only 1.76 (95% CI 1.3–2.0); and the AUC was lower (0.58 ± 0.05) with a sensitivity and a specificity of 53.8% and 67.9%, respectively.

### Sensitivity and confidence of radiologists for perforation location

Radiologists scored a higher sensitivity for estimating the perforation location when the upper GIT (stomach, duodenum, jejunum and ileum) and the lower GIT (ascending, transverse, descending and sigmoid colon with rectum) were pooled together (sensitivity = 0.91 ± 0.04). When they had to guess the individual parts of the GIT, the sensitivity dropped to 0.68 (± 0.09) at a relatively high confidence level of 3.6 (± 0.2), meaning that the radiologists were “reasonably sure” of their location prediction (Table 2).

**Table 2:** Sensitivity of the radiologists for perforation location

	SENS 1–8	SENS (up/lo GIT)	Confidence GIT 1–8
R1	0.75	0.93	3.8
R2	0.59	0.89	3.8
R3	0.61	0.86	3.4
R4	0.79	0.96	3.6
All	0.68 (± 0.09)	0.91 (± 0.04)	3.6 (± 0.2)

*R* reader, *SENS* sensitivity, *sd* standard deviation, *up/lo GIT* upper/lower gastrointestinal tract

### Volumetry (man versus machine) and interobserver agreement among radiologists

**Fig. 10:**
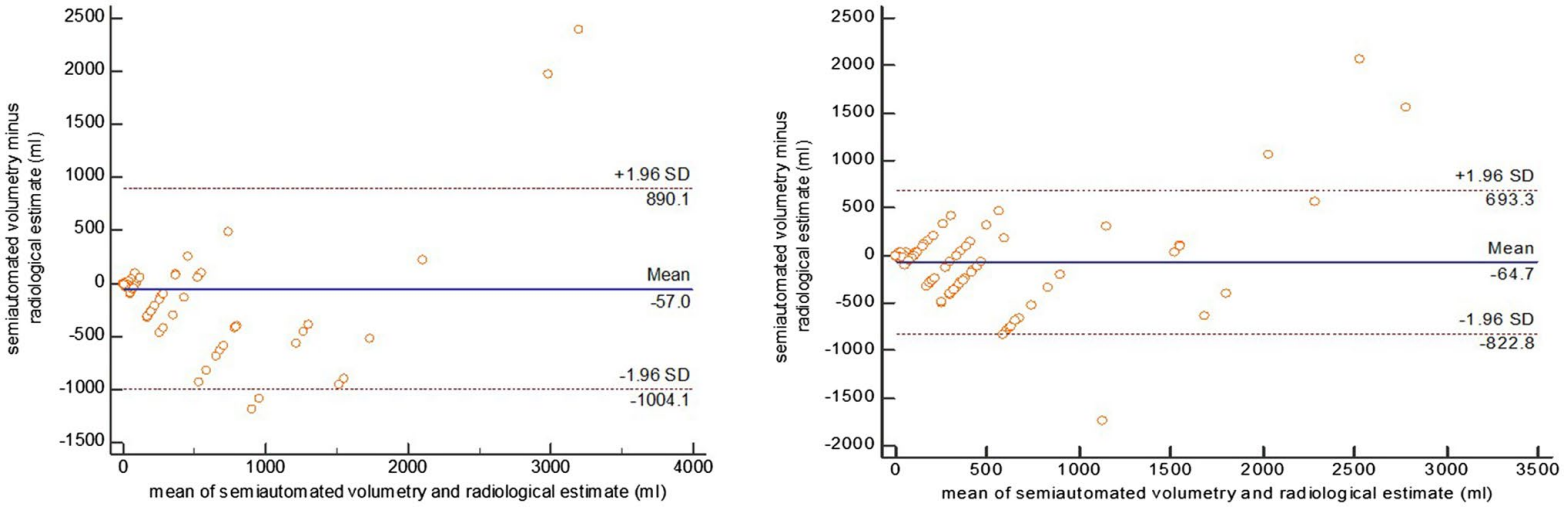
Bland–Altman plots: volume differences between machine and radiologist for air (left side) and ascites (right side)

The correlation coefficients (CCs) between semiautomated volume measurements and volume estimates of raters 1, 2, 3, and 4 were 0.96 (CI 0.93 to 0.98), 0.75 (CI 0.53 to 0.87), 0.82 (CI 0.67 to 0.91) and 0.91 (CI 0.81 to 0.95) for ascites, respectively, with a pooled coefficient for all raters of 0.80 (CI 0.72 to 0.85). For air volume measurement, the CC between the machine and the radiologists was 0.87 (CI 0.76 to 0.94), 0.75 (CI 0.55 to 0.86), 0.78 (CI 0.58 to 0.89), and 0.85 (CI 0.71 to 0.93), with a pooled CC of 0.80 (CI 0.72 to 0.85). On average, radiologists measured 57 ± 474 ml more air and 64 ± 379 ml more ascites for each case compared to the ground truth (semiautomated volumetry, Fig. 10).

The interobserver agreement between rater 1 and rater 2 for the volume of free air was 0.69 ± 0.05, and their interobserver agreement for the volume of ascites was 0.55 ± 0.05. The agreement on the perforation location was 0.81 ± 0.04. For raters 3 and 4, the agreements for air volume, ascites volume and perforation location were 0.64 ± 0.05, 0.68 ± 0.04, and 0.81 ± 0.06, respectively. Overall, substantial agreement between air and ascites estimates by the naked eye was reached (0.64 ± 0.05), and for the perforation location, almost perfect agreement could be scored (0.81 ± 0.05). There was no difference in correct classification between the more experienced radiologists (1/2) and moderately experienced radiologists (3/4) (*p* = 0.4).

## Discussion

Our results demonstrate that both ascites and free air volumes are larger in upper GIT perforations. Radiologists could very accurately name the location of the perforation using their experienced skills for detection of the smallest air bubbles around the perforation or the detection of wall defects. Experienced radiologists are advantageous, but for residents and fellows on night shifts in an emergency room, determining the amount of ascites may help to find the perforation location, which may be very useful for visceral surgeons.

Determining the volume of ascites might boost the confidence level of radiologists in finding the perforation location. With the application of a simple rule, perforation locations can be classified as upper or lower GIT. The rule says that one must estimate whether the ascites volume is more or less than 333 ml, which is the exact volume of a soda can. This approach helps radiologists image and compare liquid volumes. In comparison to the semiautomated volume measurement, the radiologists reached a high agreement with the machine that was comparable to the agreement of one radiologist with another. However, the radiologists estimated the volumes to be slightly higher (by 55–65 ml). Since the largest amount of free air is seen in perforations at the beginning and at the end of the GIT (stomach, descending and sigmoid colon) and ascites is found more in the presence of upper GIT perforations, the ratio of the sum or the delta of air and ascites is obviously not as helpful as the amount of ascites alone for localizing the perforation.

The two most frequent sites of perforation were sigmoid colon (32.6%) and stomach (20.3%), combined they represented more than 50% of all our study cases. With this information alone radiologists should know where to start looking for a leak in the GIT. The fact that perforations in the lower GIT demonstrated less amount of ascites, helps separating the two locations. It needs to be indicated that there was an overlap between air and fluid volume between the stomach and the sigmoid colon as shown in Figs. 4 and 5. Therefore, sensitivity and AUC of the proposed volume cutoffs were never 100% for classifying the perforations into upper and lower GIT perforations.

**Fig. 11:**
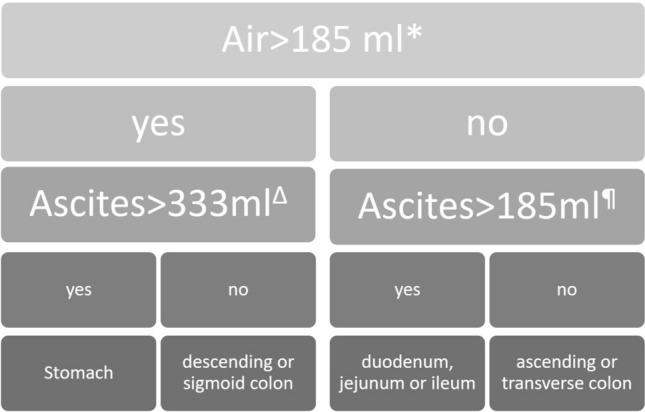
Perforation location flow chart. *A cutoff value of air volume > 185 ml led to a sensitivity, specificity and AUC of 56.1, 73.0 and 0.64 ± 0.04 for classification into stomach, descending or sigmoid colon perforations. ^Δ^The threshold of ascites > 333 ml then demonstrated a sensitivity, specificity and AUC of 51.4, 79.4 and 0.69 ± 0.05 for distinguishing stomach versus descending or sigmoid colon perforations, respectively. ^¶^Ascites volumes over 185 ml could classify the middle GIT into the duodenum, jejunum or ileum with a sensitivity, specificity and AUC of 58.7, 71.4 and 0.59 ± 0.07, respectively

In the future, the following two-step algorithm needs to be investigated (Fig. 11):

1. The amount of free air determines whether the perforation is located in the middle of the GIT or not.
2. The volume of ascites then determines if the perforation location is in the upper or lower GIT.

When the classification of the radiologists was pooled into the 4 segments suggested by the 2 step algorithm (Fig. 11), the four readers together misclassified the perforation location in 69 cases. In these cases the readers demonstrated low confidence, and the proposed algorithm detected 28 correct locations of perforation (= 40.6%, *p* value was 0.0458 compared to chance (25%)). Previous reports have emphasized CT manifestations of bowel perforation secondary to various causes. However, no previous reports have tried to quantify the most useful findings as free air and fluid. For example, Ongolo-Zogo et al. [27] reported on a series of 10 perforated gastroduodenal peptic ulcers in which two important CT findings were indicative of the site of perforation: discontinuity in the bowel wall in six patients and tiny extraluminal air bubbles in close proximity to the bowel wall in two patients. Miki et al. [28] also reported direct visualization of a ruptured colonic wall in four of six patients with colonic perforation. In the study of Hainaux et al. [21], the authors concentrated on free air bubbles in close proximity to the bowel wall and segmental bowel wall thickening as strong predictors of perforation at the site.

Our approach differs from that in the study of Seishima et al. [29], in which the author retrospectively concentrated on the CT attenuation values of ascites, demonstrating a higher density of ascites in patients with colorectal perforation than in those with perforations at other sites.

The study of Shanmuganathan et al. [30] shows that helical CT with administration of rectal, oral, and IV contrast material is highly accurate for evaluating patients with penetrating injuries to the thoracoabdominal region. Nevertheless, only 15% of patients with bowel injury showed oral or rectal contrast material extravasation. In our study, we focused mainly on patients with nontraumatic bowel perforations. In such patients, it is often difficult to obtain opacification of the ascites, future studies will have to investigate the density of ascites and perforation location in traumatic and nontraumatic patients. We halfway confirmed the hypothesis that upper GIT perforations yield more free air, but we had to reject the hypothesis that lower GIT perforations would result in more ascites. Our results showed the exact opposite. Our aim was to devise a simple rule that can always be applied without complicated time-consuming measurements at every emergency CT imaging unit, based on the fact that retrospective identification of the site of perforation helps the emergency department physician plan the appropriate treatment in a potentially unstable patient and assists the surgeon in planning the correct surgical approach.

### Limitations

We did not consider the delay between the time of onset of symptoms and the time of CT examination. Potential peritonitis caused by either upper or lower GIT perforations may lead to more ascites over time, which could confound our results. Our results represent the measurements in a consecutive population of perforation patients in a tertiary care hospital center. Factors other than location are not included in this study such as size of the perforation, density of ascites or whether the location was intraperitoneal or extraperitoneal. We wanted to focus on the many cases where the location could not primarily be identified by a large interruption of the bowel wall or by small gas bubbles around an extraperitoneal or covered perforation.

## Conclusions

Our results demonstrate that the amount of free air is larger in upper GI and distal lower GI perforations than in other sites of the gut, and upper GI perforations have a greater volume of ascites. Using a two-step process based on the volumes of free air and free fluid can accurately localize the site of perforation to the upper or lower GI tract. When used in conjunction with other CT findings, such as location of small extraluminal gas bubbles, these findings can increase the confidence of the radiologist in identification of the site of bowel perforation. This algorithm may be especially helpful for residents or junior attendings in diagnosing the perforation site.

Such information is of vital assistance to the visceral surgeon.

## Data Availability

Data are available upon special request.
